# Optimal Energetic-Trap Distribution of Nano-Scaled Charge Trap Nitride for Wider *V_th_* Window in 3D NAND Flash Using a Machine-Learning Method

**DOI:** 10.3390/nano12111808

**Published:** 2022-05-25

**Authors:** Kihoon Nam, Chanyang Park, Jun-Sik Yoon, Hyeok Yun, Hyundong Jang, Kyeongrae Cho, Ho-Jung Kang, Min-Sang Park, Jaesung Sim, Hyun-Chul Choi, Rock-Hyun Baek

**Affiliations:** 1 Department of Electrical Engineering, Pohang University of Science and Technology (POSTECH), Pohang 37673, Korea; namgee4970@postech.ac.kr (K.N.); chypark@postech.ac.kr (C.P.); junsikyoon@postech.ac.kr (J.-S.Y.); myska315@postech.ac.kr (H.Y.); hdjang@postech.ac.kr (H.J.); krcho5252@postech.ac.kr (K.C.); 2 SK hynix Inc., Cheongju-si 28429, Chungcheongbuk-do, Korea; hojung.kang@sk.com (H.-J.K.); jaesung1.sim@sk.com (J.S.); 3 SK hynix Inc., Icheon-si 17336, Gyeonggi-do, Korea; minsang.park@sk.com; 4 Department of Electrical Engineering, Yeungnam University, Gyeongsan 38541, Korea; pogary@ynu.ac.kr

**Keywords:** 3D NAND Flash, charge trap nitride, gradient-descent method, machine learning, multi-level cell, trap distribution, threshold voltage window

## Abstract

A machine-learning (ML) technique was used to optimize the energetic-trap distributions of nano-scaled charge trap nitride (CTN) in 3D NAND Flash to widen the threshold voltage (*V_th_*) window, which is crucial for NAND operation. The energetic-trap distribution is a critical material property of the CTN that affects the *V_th_* window between the erase and program *V_th_*. An artificial neural network (ANN) was used to model the relationship between the energetic-trap distributions as an input parameter and the *V_th_* window as an output parameter. A well-trained ANN was used with the gradient-descent method to determine the specific inputs that maximize the outputs. The trap densities (*N_TD_* and *N_TA_*) and their standard deviations (*σ_TD_* and *σ_TA_*) were found to most strongly impact the *V_th_* window. As they increased, the *V_th_* window increased because of the availability of a larger number of trap sites. Finally, when the ML-optimized energetic-trap distributions were simulated, the *V_th_* window increased by 49% compared with the experimental value under the same bias condition. Therefore, the developed ML technique can be applied to optimize cell transistor processes by determining the material properties of the CTN in 3D NAND Flash.

## 1. Introduction

In recent years, solid-state drives (SSDs) have been developed to satisfy the requirements for rapidly storing and using large amounts of data. NAND Flash is a key and widely used component in SSDs because of its suitability for mass production and reasonable bit cost [1]. NAND Flash has been developed with innovation in three-dimensional (3D) architecture and charge trap nitride (CTN) as a storage node [2,3,4,5]. Further, the bit cost of NAND Flash has been reduced using multi-level cell (MLC) technology, which stores multiple bits in each single cell [6].

An MLC creates more threshold-voltage (*V_th_*) states in one cell by subdividing the amount of charge injected into the storage node. As more *V_th_* states are created, the number of bits stored per cell increases, and therefore, the bit cost considerably reduces [7,8]. Unfortunately, the number of sections with overlaps between subdivided *V_th_* states also increases, making it difficult to distinguish the actual *V_th_* states and thereby degrading the device reliability [9,10,11].

Multiple *V_th_* states are created within a limited *V_th_* window between the erase (ERS) and the program (PGM) *V_th_* [12,13]. Then, if the *V_th_* window is widened, the errors in distinguishing *V_th_* states can be reduced by obtaining the margin of each *V_th_* state. In the previous works, wide *V_th_* windows were achieved by adopting new materials and bias conditions [14,15]. However, qualitative analyses for improving the *V_th_* window have not sufficiently focused on the material properties of the storage node.

The energetic-trap distribution is an important material property of the CTN, a nano-scaled thin film in 3D NAND Flash. Previously, it was discussed based on the analytical model for charges [16]. The energetic-trap distribution can be extracted using retention models [17] and trap spectroscopy by charge injection and sensing (TSCIS) [18]. Furthermore, the energetic-trap distributions are determined by the gas flow ratio deposited on the CTN [19,20]. These studies indicated that energetic-trap distributions are controllable and that their profiles determine the ERS *V_th_* (*V*_*th*,*ers*_) and PGM *V_th_* (*V*_*th*,*pgm*_) [17,21]. We tried to maximize the *V_th_* window, that is, the sum of the absolute value of *V*_*th*,*ers*_ (|*V*_*th*,*ers*_|) and *V*_*th*,*pgm*_, by optimizing the energetic-trap distributions.

We also used a novel machine-learning (ML) method to improve the *V_th_* window. Recently, ML has been used for predicting and optimizing nanoscale transistors [22,23,24]. Moreover, it can help quantitatively determine the complex material properties of the CTN with high speed and accuracy. Therefore, an artificial neural network (ANN) [25] was trained by modeling eight inputs that determine the energetic-trap distributions and the two outputs |*V*_*th*,*ers*_| and *V*_*th*,*pgm*_. Next, the energetic-trap distributions were investigated to realize a large *V_th_* window by the gradient-descent method, a widely used optimization algorithm.

In this study, the ML method helps determine the optimized energetic-trap distributions and thereby control the material properties of the CTN in 3D NAND Flash. We quantitatively found the energetic-trap distributions that resulted in the largest *V_th_* window. The remainder of this paper is organized as follows. In Section 2, the simulation data and ML method used to train the ANN are introduced. In Section 3, we discuss the results of training and optimization of ML-based analysis. Finally, the conclusions are drawn in Section 4.

## 2. Simulation and Machine-Learning Method

We obtained data from TCAD simulations for training the ANN [26]. Shockley–Read–Hall recombination, drift-diffusion transport, mobility (high-field, doping and interface dependent) and Hurkx band-to-band tunneling models were adopted for the poly-Si channel. Furthermore, the nonlocal tunneling model was used to describe charge transport in ERS/PGM operations.

**Figure 1:**
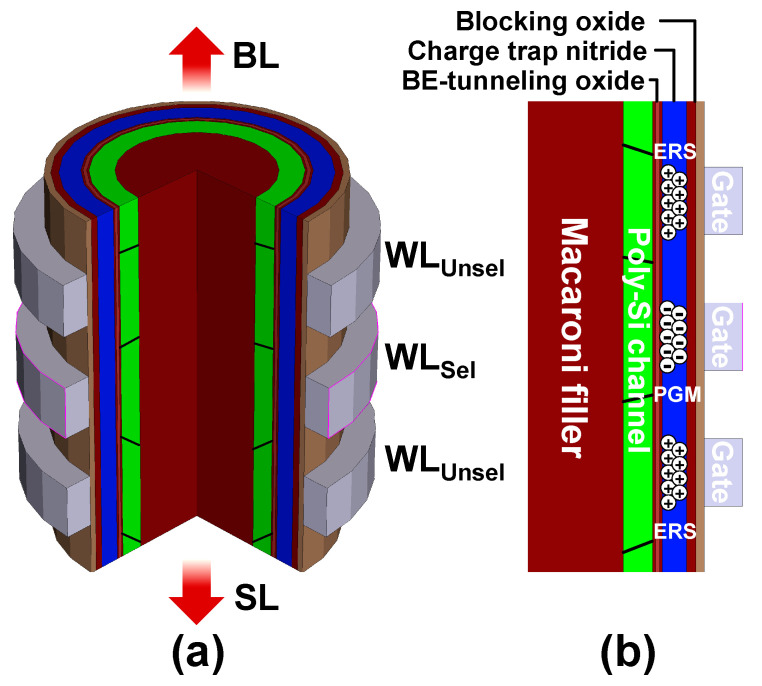
(**a**) Schematic diagram of the cylindrical structure of 3D NAND Flash. (**b**) Half cross-sectional view of string and cell stack. Depending on whether holes or electrons are present in the CTN, the state is ERS or PGM.

Figure 1 shows the schematic diagrams of 3D NAND Flash used in the simulation. Figure 1a shows a part of the 3D NAND Flash string. It has a cylindrical structure, and both ends are connected by a bit line (BL) and a source line (SL). *V_th_* is extracted from the BL current (*I_BL_*) of 1 μA vs. selected word line (WL_Sel_) voltage in the middle of the string. Figure 1b shows the half cross-sectional view of the cylindrical structure and a gate stack. Furthermore, ERS (PGM) state is the condition when holes (electrons) are filled in the CTN. The simulation structure was derived from the manufactured device [27].

**Figure 2:**
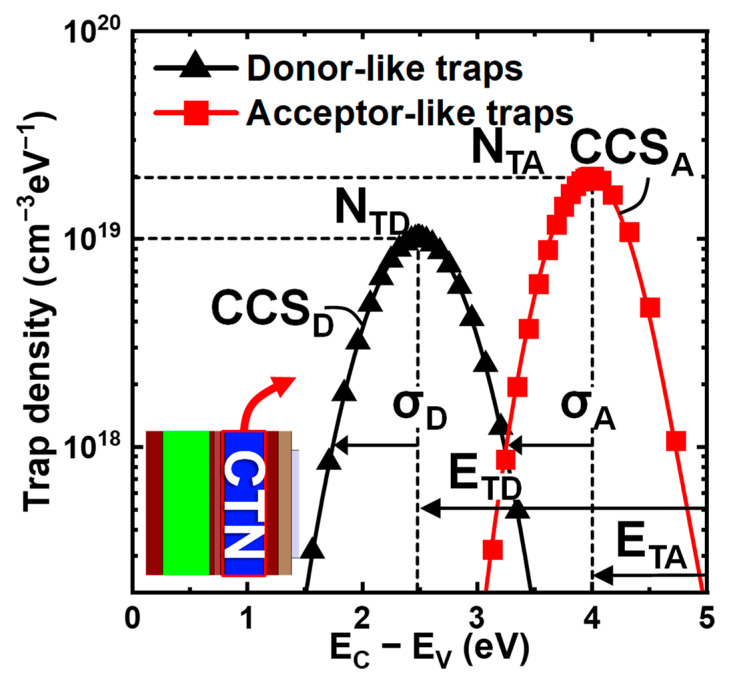
Two energetic-trap distributions comprising donor- and acceptor-like traps of the CTN. The peak energy levels (*E_TD_* and *E_TA_*) are from the conduction band (*E_C_*). Their profiles determine the *V_th_* window.

Figure 2 shows two energetic-trap distributions following the Gaussian distribution of the bandgap in the CTN. Donor- and acceptor-like traps capture holes and electrons, respectively. Each distribution consisted of the trap densities (*N_TD_* and *N_TA_*), peak energy levels (*E_TD_* and *E_TA_*), capture cross sections (*CCS_D_* and *CCS_A_*), and standard deviations (*σ_D_* and *σ_A_*). These eight inputs determine the profile of the distributions, and *V*_*th*,*ers*_ and *V*_*th*,*pgm*_ change accordingly.

**Figure 3:**
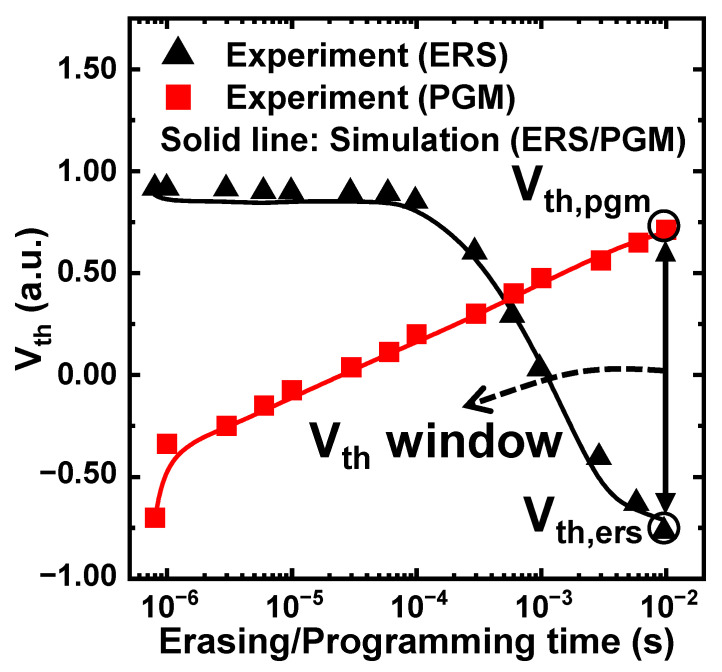
*V_th_* vs. operating time started from different ERS/PGM states during a single pulse operation. The simulation (solid line) and the experimental (symbol) data are in good agreement. The *V_th_* window is the sum of |*V*_*th*,*ers*_| and *V*_*th*,*pgm*_ at time = 10^−2^ s.

Figure 3 shows *V_th_* with the ERS/PGM operating time. Each operation was performed by applying a single pulse; the PGM operation started from the ERS state, whereas the ERS operation started from the PGM state. Under the same bias condition (*V_PGM_* = 15 V, *V_ERS_* = 20 V), the simulation data (solid lines) were calibrated with the experimental data (symbols) by adjusting the energetic-trap distributions of the CTN. The calibrated simulation data were then used as training data for the ML-based analysis. In this study, the *V_th_* window is the sum of |*V*_*th*,*ers*_| and *V*_*th*,*pgm*_ at time = 10^−2^ s. The corresponding *V_th_* window contains the difference between the ERS and the highest PGM *V_th_* states of an MLC.

**Figure 4:**
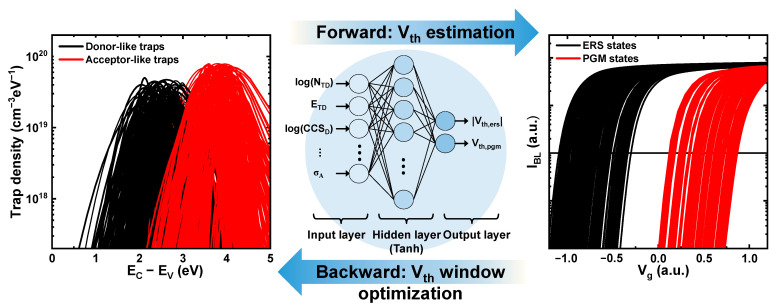
Scheme of eight inputs (Table 1) for two outputs (|*V*_*th*,*ers*_| and *V*_*th*,*pgm*_) in ANN. An MLP was used as a learning algorithm to train the ANN. In the backward direction, the eight inputs can be found in the direction of making the two outputs large.

Figure 4 shows a schematic of the ANN. First, datasets are produced from TCAD simulation for 3D NAND Flash cell. These extracted datasets are used to design the ANN. A multilayer perceptron (MLP), in which several layers of perceptrons are sequentially attached, was used for training [28]. MLP is suitable for solving nonlinear functions that cannot be solved using a single-layer perceptron, so it is useful for the training complex model. In this study, this feedforward network contains one hidden layer with 15 nodes and tanh as an activation function. In addition, it was trained using MATLAB to model the inputs and outputs [29]. The Levenberg–Marquardt method was used for the backpropagation of training, and the cost function was calculated by the mean squared error (MSE). The MSE indicates the accuracy of ML training. The smaller it is, the better is the training of the ANN. After training, the gradient-descent method was used in the backward direction to determine the optimal inputs that resulted in a large *V_th_* window. This method is widely used for finding the minimum value of the cost function in ML. Finally, we set the cost function in the direction of making large outputs. In summary, we trained the ANN using well-calibrated simulation data and then used the well-trained ANN to determine the optimal inputs that resulted in the largest outputs.

Table 1 summarizes the calibrated values and ranges of the eight inputs for training and optimizing of the ANN. These inputs were randomized within each range uniformly, and different |*V*_*th*,*ers*_| and *V*_*th*,*pgm*_ values were derived, accordingly. *N_TD_*, *N_TA_*, *CCS_D_*, and *CCS_A_* were logged, and then all parameters were standardized to improve the prediction accuracy of the ANN. In addition, there is no correlation between inputs. The calibrated values are generally the median of each range, and the entire range is reasonable [27,30]. Other material properties of the CTN were fixed only to verify the effect of energetic-trap distributions.

## 3. Results and Discussion

**Figure 5:**
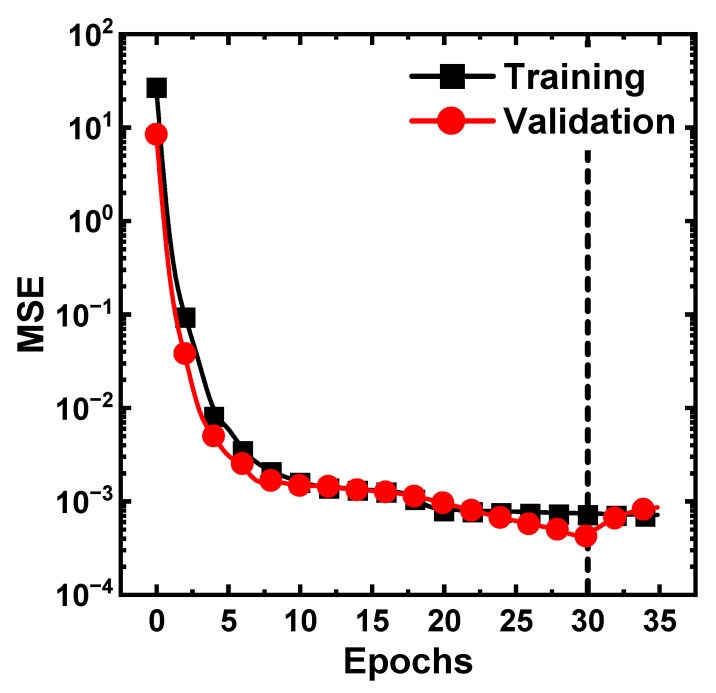
Example of MSE vs. epochs in the training and validation sets. MSEs were used as a measure of training. The model adopted five-fold cross validation later used to evaluate the test sets.

**Figure 6:**
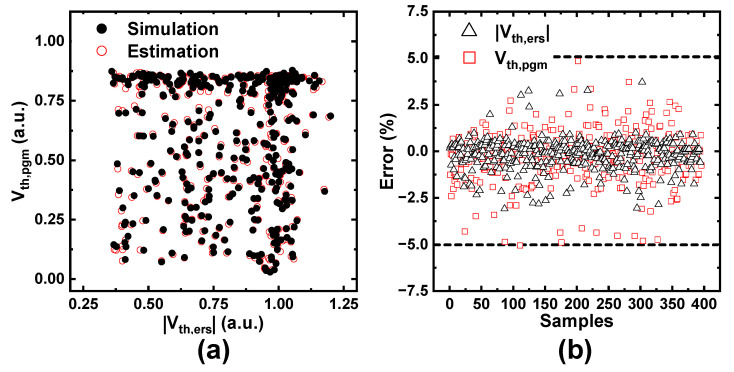
(**a**) Comparisons of |*V*_*th*,*ers*_| and *V*_*th*,*pgm*_ between simulated (closed, black) and estimated (open, red) data, and (**b**) errors are within ±5% in the test sets. MSEs are 0.7368 × 10^−3^ and 0.8472 × 10^−3^, respectively.

Figure 5 shows the example of MSEs in the training and validation sets with epochs. Here, epoch refers to the number of times the entire data have passed through the neural network. A total data of 1980 samples were used for ML training. First, they were used for training and test sets with a weight of 80/20. Then, the training sets were divided five-fold. Each fold became a validation set once, and the mean value of the five evaluations was used to determine the performance of the corresponding model. We also repeated this entire process five times by splitting the nodes of the hidden layer to increase the reliability of the model. As a result, the 15 nodes of the hidden layer have the lowest MSE, and the results of applying the model to test sets are shown in Figure 6. Furthermore, the model was well-generalized with no overfitting and underfitting because of the five-fold cross validation.

Figure 6a compares the simulated and the estimated values of |*V*_*th*,*ers*_| and *V*_*th*,*pgm*_ in the test sets. For one input set, two outputs appear simultaneously, and the estimation is expected to be good as the two symbols overlap. Figure 6b shows the errors in the individual values. Each error is extremely small and is within the acceptable range of ±5%. Furthermore, each MSE is 0.7368 × 10^−3^ and 0.8472 × 10^−3^; therefore, the prediction accuracy is very high. These results confirmed that the ANN was trained well. Furthermore, the ANN model was superior in learning speed and accuracy in our datasets compared with other regression models, the random forest, etc.

**Figure 7:**
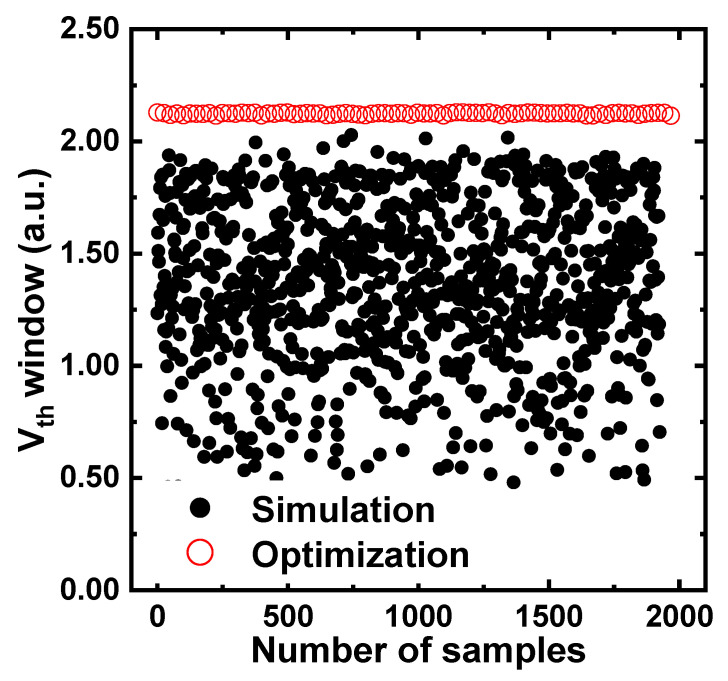
Comparison of optimized values (open, red) and simulated values (closed, black) of *V_th_* window. A large value means that the *V_th_* window is widened.

Figure 7 shows the raw simulated and optimized values of the *V_th_* window; the latter are all larger than the former. We set the cost function in the direction of maximizing the *V_th_* window. The gradient-descent method was used to determine the slope of the cost function. After setting the random inputs within the range listed in Table 1, the estimated *V_th_* window was derived using the well-trained ANN. Then, the random inputs were set again until the slope reached the extreme value. Finally, the inputs that resulted in a large *V_th_* window were found. Table 2 lists the optimized inputs that resulted in the largest *V_th_* window after 2000 iterations.

**Figure 8:**
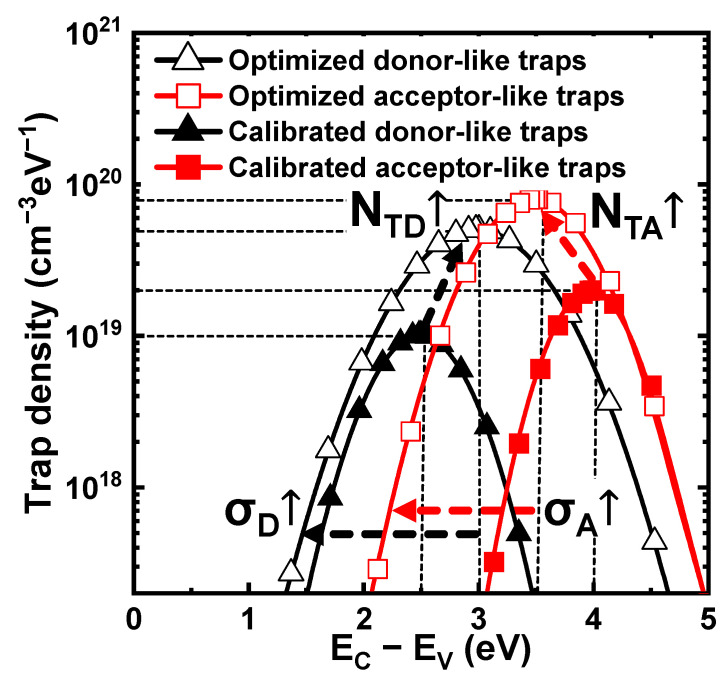
Comparison of profiles between optimized (open) and calibrated (closed) energetic-trap distributions. *N_TD_*, *N_TA_*, σ_D_, and σ_A_ strongly influence the *V_th_* window.

Figure 8 compares the calibrated and the optimized energetic-trap distributions. The ML results indicated that *N_TD_*, *N_TA_*, *σ_TD_*, and *σ_TA_* strongly influenced the *V_th_* window. When they increased, both |*V*_*th*,*ers*_| and *V*_*th*,*pgm*_ increased significantly. This is because many available trap sites could capture more holes or electrons. When *E_TD_* decreased and *E_TA_* increased, both |*V*_*th*,*ers*_| and *V*_*th*,*pgm*_ increased slightly. This is because the deeper energy of each distribution reduced the attempt-to-escape factor [31]. However, *E_TD_* and *E_TA_* mainly determined the retention characteristics; therefore, the correlation with the *V_th_* window was weak. Similarly, *CCS_D_* and *CCS_A_* had small effects on the *V_th_* window. In summary, large *N_TD_*, *N_TA_*, *σ_TD_*, and *σ_TA_* resulted in a large *V_th_* window owing to the availability of a larger number of trap sites. However, *E_TD_*, *E_TA_*, *CCS_D_*, and *CCS_A_* had a small correlation with the *V_th_* window because we did not consider the retention characteristics.

**Figure 9:**
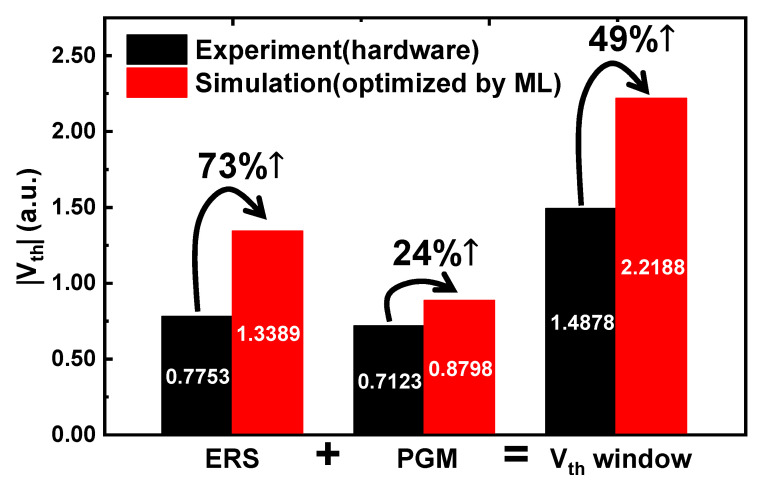
*|V_th_|* values in ERS/PGM operations and *V_th_* window. Values estimated using ML outperform experimental values overall.

Figure 9 shows the experimental and simulated *|V_th_|* values optimized by ML. Here, the simulated *V_th_* window was calculated from the best inputs in Table 2. In this case, the *|V_th_|* error between the simulation and the ANN was within 4.34%. Therefore, the simulated *V_th_* windows were reliable. The *V_th_* window increased by 49%. In other words, the *V_th_* window can be sharply increased by optimizing the energetic-trap distributions. Therefore, we can provide a guideline for maximizing the *V_th_* window, although the precise process remains difficult.

## 4. Conclusions

ML-based analysis was used to obtain optimized energetic-trap distributions for the CTN in 3D NAND Flash to improve the *V_th_* window. The ANN enables modeling the relationship between eight inputs that determine the energetic-trap distributions and the two outputs, |*V*_*th*,*ers*_| and *V*_*th*,*pgm*_. The ANN was trained using well-calibrated simulation data with experiments, and the MSEs were found to be small. Then, we used the gradient-descent method to determine the best inputs that resulted in the largest *V_th_* window. *N_TD_*, *N_TA_*, *σ_TD_*, and *σ_TA_* significantly influenced the *V_th_* window. As they increased, the *V_th_* window grew because of the large number of trap sites. In particular, when the best inputs obtained using ML were employed, the *V_th_* window increased by 49% compared with the experimental value. This study should enable the determination of the *V_th_* window from the material properties of the CTN in 3D NAND Flash. More generally, this work implies that ML can help to solve the complex problem of nanomaterials accurately and optimize it rapidly.

## Figures and Tables

**Table 1:** Input parameters of energetic-trap distributions of the CTN.

Trap Parameter	Min	Max	Calibrated
Density of donor-like traps, *N_TD_* (cm^−3^·eV^−1^)	5.00 × 10^18^	5.00 × 10^19^	1.00 × 10^19^
Peak energy level of donor-like traps, *E_TD_* (eV)	2.00	3.00	2.50
Capture cross section of donor-like traps, *CCS_D_* (cm^2^)	1.00 × 10^−15^	1.00 × 10^−11^	1.00 × 10^−13^
Standard deviation of donor-like traps, *σ_D_* (eV)	0.10	0.50	0.35
Density of acceptor-like traps, *N_TA_* (cm^−3^·eV^−1^)	8.00 × 10^18^	8.00 × 10^19^	2.00 × 10^19^
Peak energy level of acceptor-like traps, *E_TA_* (eV)	0.80	1.50	1.00
Capture cross section of acceptor-like traps, *CCS_A_* (cm^2^)	1.00 × 10^−15^	1.00 × 10^−11^	1.00 × 10^−13^
Standard deviation of acceptor-like traps, *σ_A_* (eV)	0.10	0.50	0.30

**Table 2:** Optimized input parameters of energetic-trap distributions of the CTN.

Trap Parameter	Value
*N_TD_* (cm^−3^·eV^−1^)	5.00 × 10^19^
*E_TD_* (eV)	2.00
*CCS_D_* (cm^2^)	1.00 × 10^−15^
*σ_D_* (eV)	0.50
*N_TA_* (cm^−3^·eV^−1^)	8.00 × 10^19^
*E_TA_* (eV)	1.45
*CCS_A_* (cm^2^)	1.00 × 10^−15^
*σ_A_* (eV)	0.50

## Data Availability

Not applicable.
